# A dramatic course after the resection of an SMARCA4‐deficient undifferentiated tumour

**DOI:** 10.1002/rcr2.1001

**Published:** 2022-07-06

**Authors:** Takafumi Iguchi, Shinjiro Mizuguchi, Kyukwang Chung, Ryu Nakajima, Makoto Takahama

**Affiliations:** ^1^ Department of General Thoracic Surgery Osaka City General Hospital Osaka Japan

**Keywords:** histology/cytology, lung cancer, thoracic surgery

## Abstract

Thoracic SMARCA4‐deficient undifferentiated tumours are ordinarily found as a huge mass with systemic metastasis, and the prognosis is poor. The potential of immunotherapy for these unresectable tumours has been reported. An asymptomatic 68‐year‐old man with a smoking history had a left lung mass without distant metastasis and underwent complete resection. Two months after surgery, with no adjuvant therapy, he developed multiple distant metastases with aphasia and died 4 months after surgery. Adjuvant treatment may be necessary with immune checkpoint inhibitors, with a closer follow‐up to detect recurrence without symptoms.

## Graphical abstract

Thoracic SMARCA4‐deficient undifferentiated tumours are ordinarily found as a huge mass with systemic metastasis, and the prognosis is poor. Although a small number of operable cases have been reported, it is unclear whether complete resection contributes to a longer prognosis. Here, we present a case of SMARCA4‐UT in which distant metastasis occurred shortly after complete resection, leading to an aggressive course.

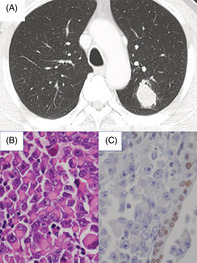

## INTRODUCTION

Thoracic SMARCA4‐deficient undifferentiated tumour (SMARCA4‐UT) is a high‐grade malignant neoplasm caused by the loss of function of SMARCA4, a key member of the SWItch/Sucrose‐NonFermentable (SWI/SNF) adenosine triphosphatase (ATPase)‐dependent chromatin remodelling complex.^1^ At diagnosis, most patients have systemic metastases, and their life expectancy is 4–7 months.^1^ Although a small number of operable cases have been reported, it is unclear whether complete resection contributes to a longer prognosis. Here, we present a case of SMARCA4‐UT in which distant metastasis occurred shortly after complete resection, leading to an aggressive course.

## CASE REPORT

**FIGURE 1:**
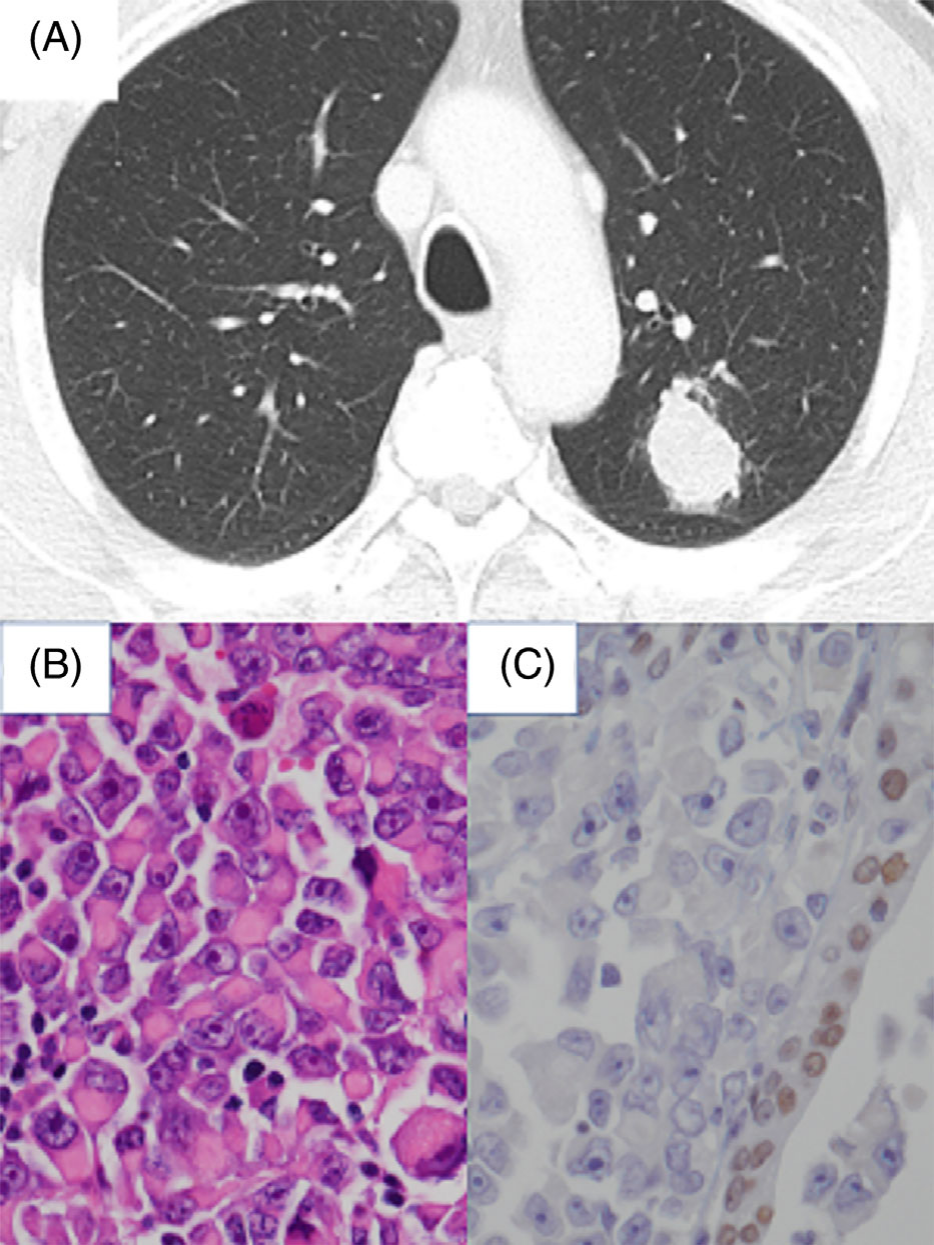
Preoperative enhanced chest computed tomography showing a tumour measuring 3.5 cm in diameter in the left upper lung lobe (A). The resected specimen showing (B) discohesive growth of rhabdoid cells and atypical cells with large nuclei and prominent nucleoli (haematoxylin and eosin [H&E]‐stained sections ×400), and (C) complete loss of SMARCA4 staining (H&E, ×400).

**FIGURE 2:**
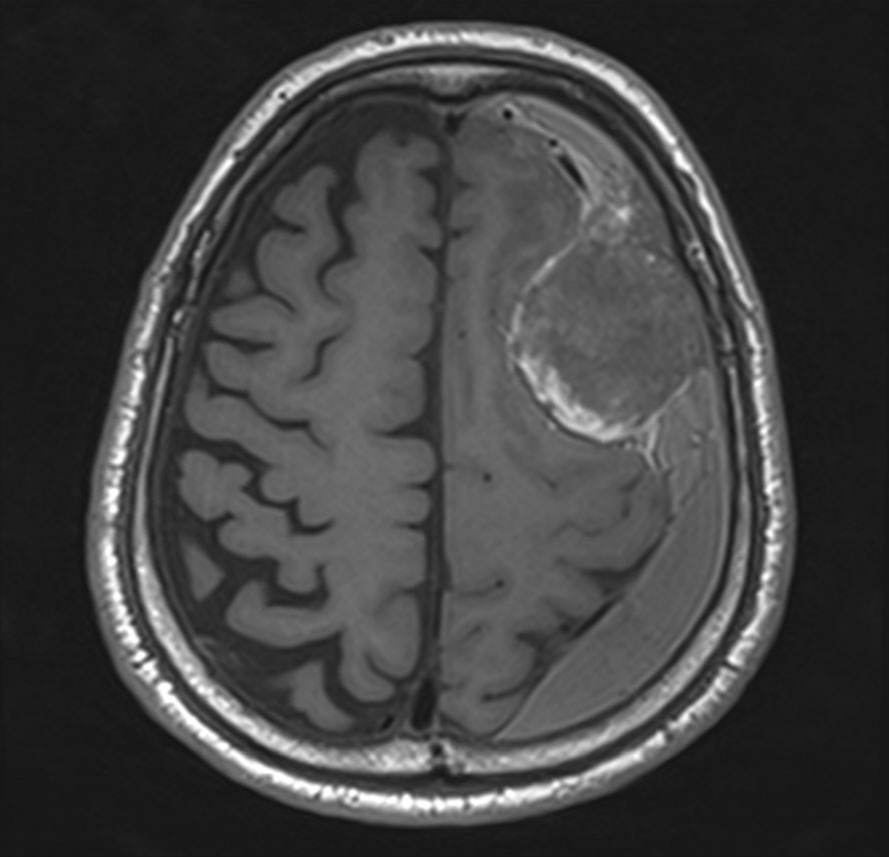
Postoperative brain magnetic resonance imaging showing intracranial metastasis 2 months after surgery.

A 68‐year‐old man with a 50‐pack‐year smoking history was referred to our hospital for evaluation of an abnormal chest radiograph. Computed tomography revealed a 3.5‐cm mass in the left upper lung lobe and an enlarged hilar lymph node (Figure 1A). The swollen lymph node had infiltrated into the left pulmonary artery (PA). Further imaging revealed no distant metastasis. Transbronchial biopsy confirmed a diagnosis of non‐small cell carcinoma (cT2aN1M0; Stage IIB). He had no symptoms, no comorbidities and a good general condition (performance status: 0; Hugh–Johns: 1). The carcinoembryonic antigen level was 3.1 ng/ml. Two weeks after the first visit, left upper lobectomy with a sleeve resection of the left upper bronchus and PA plasty was performed, and complete resection was achieved (R0). Pathology of the resected specimen revealed that the tumour measured 29 mm in diameter, with necrosis, and was composed of sheets of rhabdoid cells showing prominent nucleoli and eosinophilic cytoplasm (Figure 1B). On immunohistochemistry examinations, there was complete loss of SMARCA4 and SMARCA2 expression (Figure 1C). Other immunostaining showed thyroid transcription factor (TTF‐1) (−), p40 (−), cytokeratin (CK) 5/6 (−), AE1/AE3 (focal +), epithelial membrane antigen (EMA) (focal +), NUT (−), cluster of differentiation (CD) 34 (focal +) and integrase interactor (INI)‐1 (+). According to these findings, the tumour was diagnosed as an SMARCA4‐UT. No targetable driver mutations (epidermal growth factor receptor [EGFR], anaplastic lymphoma kinase [ALK] and ROS‐1) and a programmed death‐ligand‐1 (PD‐L1) tumour proportion score of 25%–49% were shown. The postoperative course was uneventful, and the patient was discharged on postoperative day 8. Following a review by the cancer board in our institution, no adjuvant therapies were administered because of the sarcoma component. Two months postoperatively, the patient presented to the emergency room with aphasia. Brain and abdominal imaging revealed a mass in the dura with haemorrhage, which was compressing the brain parenchyma (Figure 2), and bilateral adrenal tumours. He underwent brain tumour resection and whole‐brain irradiation (20 Gy). Although his symptoms improved temporarily, he was readmitted owing to severe pain with multiple bone metastases and gastrointestinal perforation by small bowel metastasis 1 month after the brain operation. Best supportive care was performed, and he died 4 months after the first operation. Informed consent was obtained from the patient.

## DISCUSSION

SMARCA4‐UT is histopathologically characterized by an undifferentiated or rhabdoid phenotype and SMARCA4 deficiency.^1^ This novel tumour was reported by Le Loarer et al. in 2015 as an SMARCA4 mutant neoplasm.^2^ The tumour was called ‘SMARACA4‐deficient thoracic sarcoma’ (SMARCA4‐DTS) until recently, but the currently recommended terminology is SMARCA4‐UT according to the 2021 World Health Organization (WHO) classification of thoracic tumours.^1^

The tumour often appears in the fourth and fifth decades of life, usually with a smoking history, and shows a strong male predominance (male:female ratio of 9:1).^1^ SMARCA4‐UT can occur in a variety of organs, such as the gastrointestinal tract, ovary, kidney and lung. Among thoracic SMARCA4‐UT cases, 33 of 54 cases occurred in the mediastinum and seven in the lung. Most of these tumours are huge and invade the surrounding tissues.^1^

The tumours genetically have inactivating mutations in *SMARCA4* and often have additional mutation in *TP53*, *KRAS*, *STK11* and *KEAP1*, which are common drivers of smoking‐related lung cancer. SMARCA4 is a key member of the SWI/SNF ATPase‐dependent chromatin remodelling complex, but the details of the relationship between SMARCA4 deficiency and cancer development remain unclear. SMARCA4 deficiency is observed in about 5% of conventional non‐small cell lung cancers, which should be distinguished by epithelial architecture, diffuse strong keratin expression and ancillary marker including SMARCA2.

Owing to the aggressive behaviour of these tumours, most cases present at an inoperable stage with symptoms, and are associated with a short survival time.^1^ Fortunately, the present case was identified at a limited stage with no symptoms, and we achieved complete resection. In the English literature, including our case, there are only three reports of surgery for SMARCA4‐UT without distant metastasis and complete resection was achieved in two cases.^3^, ^4^ Stewart et al. reported a patient with a 3.5‐month recurrence‐free survival after complete resection^3^; however, postoperative treatment or prognosis after complete resection of SMARCA4‐UT remains unclear.

SMARCA4‐UT is generally insensitive to cytotoxic chemotherapy; therefore, in this case, we did not administer adjuvant chemotherapy. Although three recent cases of unresectable SMARCA4‐UTs receiving combined immunotherapy, including atezolizumab, showed a strong effect (up to 17 months progression‐free survival),^5^ there are no reports on the efficacy of immune checkpoint inhibitors (ICI) as adjuvant chemotherapy. Owing to the aggressive growth of these tumours, once symptoms appear, continuing treatment is frequently difficult, with decreased patient performance status. We consider that even with complete resection, this tumour should be viewed as systemic disease. It might be necessary to consider adjuvant treatment, including ICIs, with a closer follow‐up to detect recurrence without symptoms.

## AUTHOR CONTRIBUTION

Takafumi Iguchi, Kyukwang Chung and Ryu Nakajima were involved in the conception or design of the work, and the acquisition, analysis or interpretation of data for the work. Shinjiro Mizuguchi drafted the work and revised it critically for important intellectual content. Makoto Takahama was involved in the final approval of the version to be published.

## CONFLICT OF INTEREST

None declared.

## ETHICS STATEMENT

The authors declare that appropriate written informed consent was obtained for the publication of this manuscript and accompanying images.

## Data Availability

The data that support the findings of this study are available from the corresponding author upon reasonable request.
